# Case Report: An exceptional response to a novel immunotherapy-based combination treatment for resected pancreatic ductal adenocarcinoma with recurrence to the abdominal wall

**DOI:** 10.3389/fonc.2026.1649850

**Published:** 2026-02-23

**Authors:** Thomas Enzler, Sara Tweedy, Isabella Holmes, Laura W. Lamps

**Affiliations:** 1 Department of Medicine, University of Michigan, Ann Arbor, MI, United States; 2 Department of Pathology, University of Michigan, Ann Arbor, MI, United States

**Keywords:** PDAC, cutaneous/subcutaneous metastasis, immunochemotherapy including anti-LIF, conversion of M2 to M1 macrophages, complete response

## Abstract

Pancreatic ductal adenocarcinoma (PDAC) is a difficult-to-treat cancer with a 5-year survival rate of only 13%. Intense chemotherapies are needed to control the disease. Surgical resection, if possible, is the only curative approach. However, recurrence rates are high. The inclusion of immune checkpoint inhibitors in treatment hasn’t shown benefit, most likely because of the cancer’s highly immunosuppressive tumor microenvironment (TME). Leukemia Inhibitory Factor (LIF) is a cytokine that contributes to this profound immunosuppression, which is thought to be at least partly mediated by pro-tumoral (M2-like) tumor-associated macrophages (TAMs). Those macrophages harbor LIF receptors on their surface. The addition of the anti-LIF antibody MSC-1 to a combination of anti-programmed death-ligand 1 (anti-PD-L1) and chemotherapy has shown encouraging preclinical results, successfully reversing pro-tumoral TAMs into anti-tumoral (M1-like) macrophages. Here, we report a patient with recurrent PDAC in the abdominal wall and peritoneum, that immediately went into complete remission after receiving an investigational treatment with MSC-1 and anti-PD-L1 combined with standard-of-care chemotherapy. We discuss immunological mechanisms that may have contributed to the impressive treatment response.

## Introduction

PDAC is one of the deadliest solid cancers. Even if the disease is resected and standard-of-care treatment protocols have been applied, recurrence rates are high, and, ultimately, most patients will die from the disease (1). Current chemotherapies have limited efficacy. Accumulating toxicities often require dose reductions or even omission of agents, further diminishing treatment responses. To date, immuno- or immuno-combination therapies have not been shown to significantly improve progression-free or overall survival (2). Leukemia Inhibitory Factor (LIF) is a cytokine belonging to the interleukin-6 superfamily. It is involved in diverse physiological and pathophysiological processes, including inflammation in general and immune responses to infections (3). In cancer, LIF maintains an immunosuppressive tumor microenvironment (TME) by inhibiting the influx of T lymphocytes and promoting the presence of pro-tumoral (also known as M2-like) tumor-associated macrophages (TAMs) (4). LIF also promotes epithelial-mesenchymal transition (EMT), which plays an important role in metastasis formation (5). Elevated circulating LIF levels in PDACs correlate with a poor prognosis (6). MSC-1 is a humanized anti-LIF antibody. When combined with an anti-programmed death-ligand 1 (anti-PD-L1) agent and chemotherapy, it enhanced therapeutic efficacy in preclinical PDAC models (7). These findings led to the design of an investigational regimen combining MSC-1 with anti-PD-L1 and standard-of-care chemotherapy gemcitabine/nab-paclitaxel for advanced/metastatic PDAC. Here, we report a patient with recurrent PDAC in the abdominal wall who responded exceptionally well to this novel combination treatment.

## Case presentation

A 67-year-old patient with a past medical history of type 2 diabetes mellitus, hypertension, hyperlipidemia, and gastroesophageal reflux disease presented with worsening epigastric pain starting in spring 2021. The patient was ultimately diagnosed with adenocarcinoma of the body of the pancreas in June 2021. Immunohistochemistry showed preserved mismatch repair protein expression and HER2 negativity. Next-generation sequencing revealed a KRAS G12V mutation, a TP53 loss-of-function mutation, and loss of copy number of SMAD4 and CDKN2A, all relatively common findings in PDACs. Tumor mutation burden was low. Genomic sequencing did not reveal a pathogenic germline variant.

Initial imaging was consistent with borderline resectable disease. Consequently, the patient received eight rounds of neoadjuvant standard-of-care combination chemotherapy with fluorouracil/oxaliplatin/irinotecan (FOLFIRINOX) (8). While receiving treatment, the tumor marker carbohydrate antigen (CA) 19–9 decreased from 627 U/mL to 196 U/mL. On CT scans, the patient exhibited a moderate treatment response. A distal pancreatectomy with splenectomy and portal vein resection/reconstruction was performed in October of 2021. The final pathological stage was ypT3N2, with 6 of 16 lymph nodes being positive and a positive margin. After surgery, the patient received four additional rounds of FOLFIRINOX. Given a positive margin, treatment was concluded by adding five weeks of chemoradiation with capecitabine as a radiosensitizer (9). At that time, the CA 19–9 was within normal limits (19 U/mL). The patient then entered a surveillance program and remained disease-free for 15 months.

**Figure 1:**
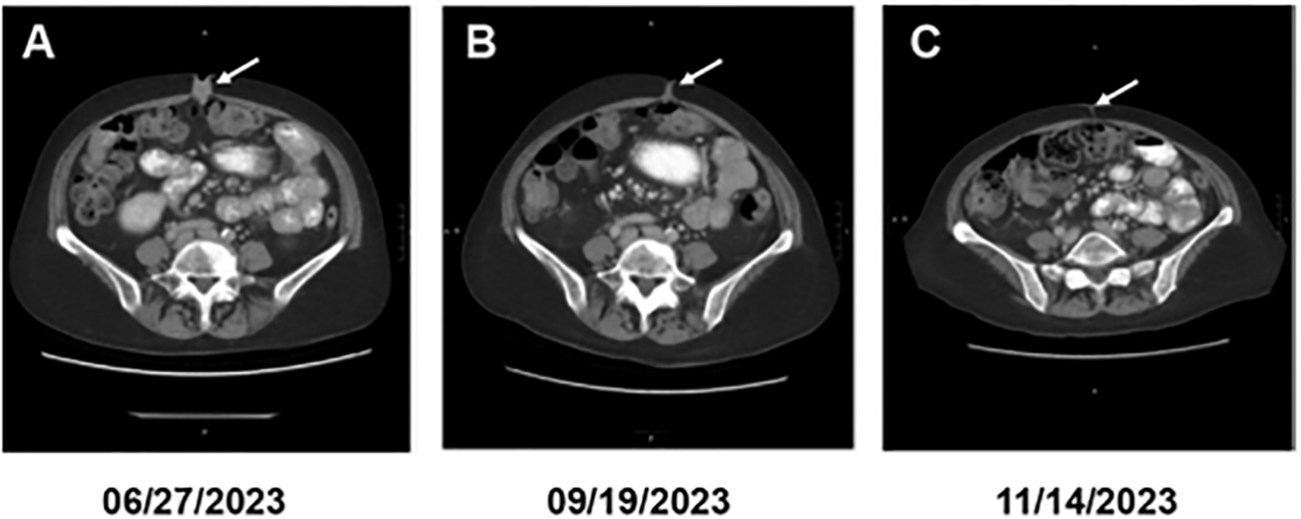
Serial CTs of the abdomen at different dates as indicated. The arrow points to the cancerous lesion (gray area) that completely disappeared on the last image.

**Figure 2:**
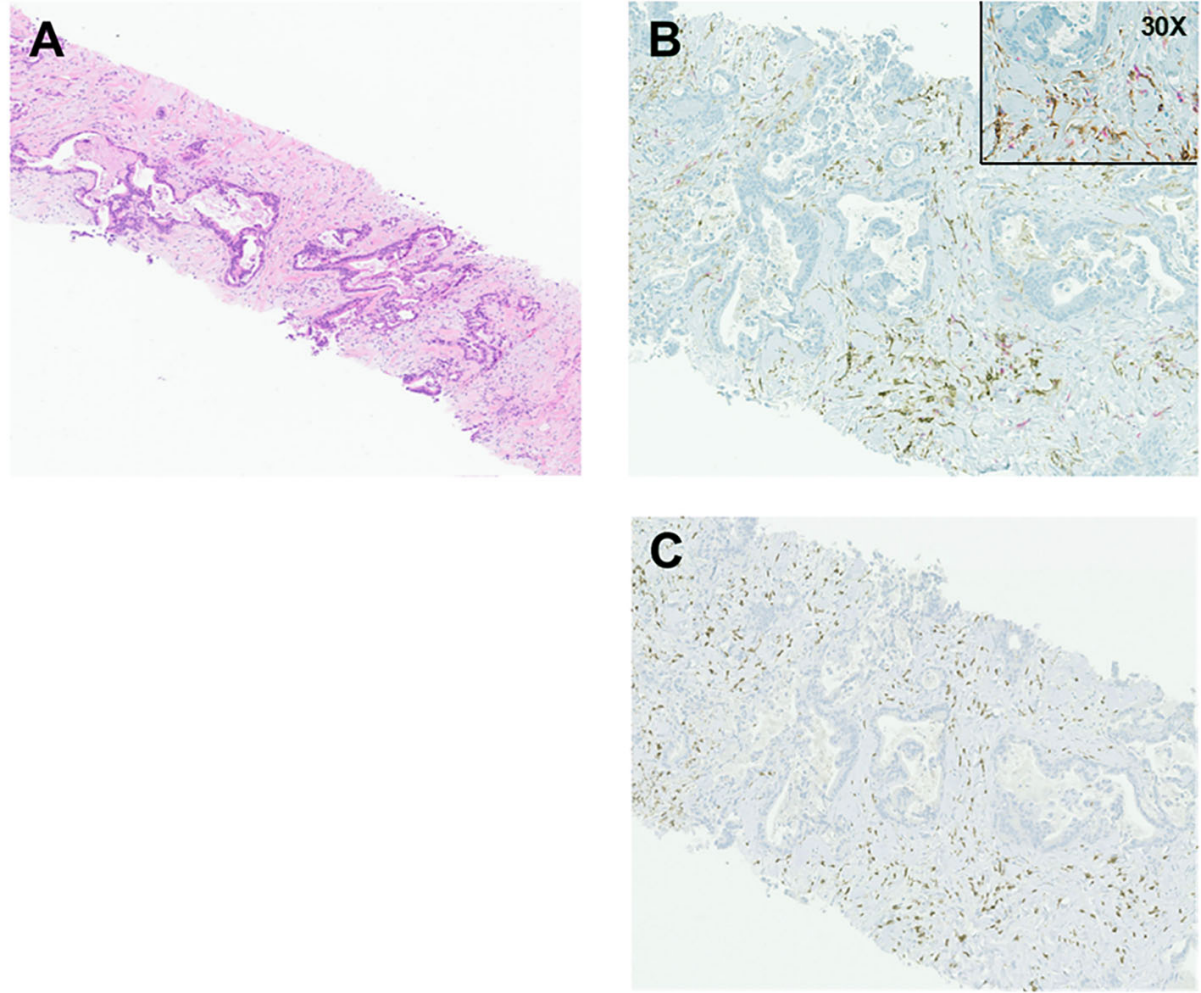
**(A)** H&E (4X). Sections demonstrate soft-tissue cores infiltrated by neoplastic cells forming irregular, jagged-shaped glands with surrounding desmoplastic stroma. **(B)** CD163 and CD4 stains (10X, and 30X as indicated). CD163^+^ macrophages are highlighted by brown cytoplasmic staining within the desmoplastic stroma, with interspersed and scattered CD4^+^ T cells highlighted by red cytoplasmic staining. Immunohistochemical stains were performed per manufacturer’s instructions on a Ventana immunostainer using a mouse monoclonal antibody for CD163 (10D6, Leica) and a rabbit monoclonal antibody for CD4 (SP35, Ventana). **(C)** CD3 stain (10X). CD3 immunohistochemical stain highlights scattered, sparse CD3^+^ T cells (brown) in the desmoplastic stroma. For the stains, we used a monoclonal rabbit anti-CD3 antibody (2GV6, Ventana).

In June of 2023, the patient started complaining of pain around the navel, where a growing lump could be palpated. CA 19–9 raised to 270 U/mL (07/31/2023). A CT abdomen and pelvis showed a 2.4 x 1.7 x 1.4 cm hyperenhancing nodule within the umbilicus (**Figure 1A**, target lesion per RECIST 1.1 (10)) and nodularity in the peritoneum (non-measurable per RECIST criteria). Biopsy of the umbilical nodule was consistent with metastatic PDAC (**Figure 2A**). Given the disease recurrence, the patient was offered an investigational protocol consisting of a combination of anti-LIF MSC-1, anti-PD-L1 durvalumab, and chemotherapy gemcitabine/nab-paclitaxel in July of 2023. Treatment protocol was as follows: MSC-1–1500 mg iv Q2W, durvalumab 1500 mg iv Q4W, gemcitabine 1000 mg/m^2^ iv, and nab-paclitaxel 125 mg/m2 iv (both on days 1,8 and 15 of a 28-day cycle). The patient was interested in participating. The patient’s performance status was excellent (ECOG 0), and there were no changes in body weight or energy level. Liver function tests and C-reactive protein levels were within normal limits. During the experimental treatment, the patient experienced some fatigue, but otherwise quality of life was at baseline. Within just two months, a CT abdomen and clinical exam demonstrated an impressive treatment response, showing a dramatic decrease in the size of the umbilical nodule to 1.7 x 0.6 x 0.4 cm (longest diameter: -30%, consistent with a partial response per RECIST) (**Figure 1B**) and of the peritoneal nodules, which were no longer visible. In November 2023, four months after treatment initiation, CA 19–9 dropped to normal limits (34 U/mL [normal <40 U/mL]), and the umbilical nodule was no longer palpable or radiologically measurable, consistent with a complete therapeutic response (**Figure 1C**). A repeat biopsy of the umbilical area showed non-specific inflammatory cells, including lymphocytes and macrophages, but no malignant cells. The patient remained in complete remission for nearly five months while continuing to receive the investigational treatment.

Unfortunately, the patient’s course was then complicated by the onset of difficult-to-manage hypertension, new shortness of breath, and worsening kidney function. A chest CT showed patchy ground-glass opacities and interstitial infiltrates. Although initially interpreted as immunotherapy-induced adverse events, it soon became clear that the overall appearance was more consistent with a Hemolytic Uremic Syndrome (HUS), likely induced by gemcitabine. This was supported by a kidney biopsy that demonstrated a focal acute and diffuse chronic thrombotic microangiopathy. The deteriorating kidney function led to the discontinuation of the treatment. Within a month, an MR abdomen and pelvis showed a new peritoneal mass, 3.6 cm in diameter, and ascites consistent with a flare-up of the cancer.

**Table 1** lists a timeline of events.

**Table 1:** Timeline of the events.

Timeline	Event	Tumor marker CA 19–9 [normal <40 U/mL]
06/2021	Diagnosis: borderline resectable PDAC	627 U/mL
06/2021 – 09/2021	Neoadjuvant treatment with FOLFIRINOX x 8 cycles	
10/2021	Surgical resection	16 U/mL
12/2021- 01/2022	Adjuvant treatment with FOLFIRINOX x 4 cycles	
02/2022-03/2022	Chemoradiation treatment (capecitabine) x 5 weeks	19 U/mL
06/2023	Recurrent disease of the abdominal wall/peritoneum. Target lesion: mass umbilicus: 2.4 x 1.7 x 1.4 cm (CT chest/abdomen/pelvis 06/27/2023)	173 U/mL
07/2023	Experimental treatment with anti-LIF, anti-PD-L1, and gemcitabine/nab-paclitaxel (start date: 07/31/2023)	270 U/mL
09/2023	Target lesion: 1.7 x 0.6 x 0.4 cm (-30% longitudinal change), disappearance of peritoneal lesions (CT chest/abdomen/pelvis 09/19/2023)	85 U/mL
11/2023	Complete radiologic and clinical response. Repeat biopsy: inflammatory cells, including lymphocytes and histiocytes/macrophages, no malignant cells (CT chest/abdomen/pelvis 11/14/2023)	34 U/mL
04/2024	Hypertension, kidney failure, pulmonary infiltrates Termination of experimental treatment on 04/15/2024	37 U/mL
05/2024	Disease recurrence in the peritoneum: new 3.1 cm lesion (MR abdomen/pelvis 05/18/2024)	98 U/mL

## Discussion

This patient’s recurrent PDAC responded remarkably well to a novel treatment consisting of anti-LIF plus anti-PD-L1 in combination with standard-of-care chemotherapy gemcitabine/nab-paclitaxel. This patient’s cancer was microsatellite stable (MSS), and the tumor mutation burden was low. There was no evidence of a DNA repair mechanism defect associated with a BRCA mutation or a mutation in the BRCA-ness pathway, which could have resulted in increased antigenicity and, thus, explained the exceptional response to immunochemotherapy (11, 12). Currently available immunotherapies or immunochemotherapies have not been effective in PDACs with intact DNA repair mechanisms (13). In a large, 1:1 randomized phase 3 study comparing gemcitabine as a single agent with gemcitabine/nab-paclitaxel in 861 patients with advanced PDAC in the first-line, the overall response rate with gemcitabine/nab-paclitaxel was 29% [95% CI, 25-34], and only one complete response was observed (14). An explanation for the exceptional treatment response seen in our patient could be the disease’s location in the abdominal wall, within the cutaneous/subcutaneous tissue. While the cutaneous/subcutaneous tissue is physiologically in a non-inflammatory state, it is particularly rich in antigen-presenting cells (APCs), including dendritic cells, macrophages, Langerhans cells (a subset of macrophages), and regulatory T cells. These cells play crucial roles in initiating or suppressing immune responses (15–17). By contrast, visceral tissue, and in particular visceral adipose tissue, is often found to be in a state of chronic inflammation with a predominance of pro-inflammatory (M1) macrophages and CD3^+^ effector T cells (18). The TME of PDAC typically comprises cancer-associated fibroblasts (CAFs) and immunosuppressive, pro-tumoral (M2) tumor-associated macrophages (TAMs), embedded in a dense acellular matrix known as the desmoplastic stroma (19). When we performed immunohistochemical stains of the cancerous tissue in the abdominal wall, we found numerous CD163^+^ cells in the desmoplastic stroma (**Figure 2B**). CD163 is a surface marker for macrophages, particularly immunosuppressive M2 TAMs, which are known to highly express LIF receptors and often express the immune checkpoint protein PD-L1 (7, 16, 20, 21). Unsurprisingly, we found only sparse numbers of CD3^+^ and CD4^+^ T cells in the TME (**Figures 2B, C**). Stimulation of macrophages with LIF increased CD163 expression and suppressed the function and proliferation of CD4^+^ and CD8^+^ T cells *in vitro* (7). We hypothesize that exposing pro-tumoral M2 TAMs to anti-LIF converted them into immunostimulatory, anti-tumoral M1 TAMs, thereby leading to an influx of CD4^+^ and CD8^+^ T cells into the TME (22). Priming of CD8^+^ T cells into anti-tumoral cytotoxic T cells and augmenting their efficacy were further enhanced by anti-PD-L1 (23). Moreover, the anti-tumoral response could have been reinforced by the pyrimidine analog gemcitabine, which is well known for its immunostimulatory properties, particularly by inducing antigenicity through its cytotoxic effect on cancer cells and by converting M2 TAMs into anti-tumoral macrophages (24).

Unfortunately, treatment response in our patient did not result in an immunologic memory strong enough to keep the cancer under control, and the disease relapsed shortly after treatment was discontinued for gemcitabine-induced HUS. Possible explanations for the rapid recurrence include insufficient T cell priming, rapid loss of antigenic stimulus after tumor clearance, or persistent systemic immunosuppression (25).

As the development of gemcitabine-induced HUS is extremely rare, there is a chance that HUS may have been triggered by the combined experimental immunochemotherapy, which the patient received. Alternatively, HUS may have been caused by immunotherapy-mediated endothelial injury (26). Thus, our case also highlights the toxicity risks associated with novel combination treatments, which necessitate close monitoring.

Results of the first-line phase 2 trial of AZD0171 (MSC-1) plus durvalumab and standard-of-care gemcitabine/nab-paclitaxel (clinicaltrials.gov; NCT04999969), in which our patient participated, were recently presented (27). 126 patients were enrolled in the study. There were no other patients with the cutaneous/subcutaneous metastatic disease. Three patients (2.4%) had a complete response, and 41 patients (32.5%) had a partial response. There was no overall survival (OS) benefit compared with historical data for gemcitabine/nab-paclitaxel (14, 27). Tumoral CD8^+^ T cell density was not associated with improved OS. Main side effects of the combination treatment were diarrhea (54.7%), nausea (54%), fatigue (42.9%), and pyrexia (42.1%). No other HUS was observed.

We understand that our proposed mechanism of treatment response in our patient has limitations, as we do not provide further experimental data, such as monitoring the composition of the TME over time, which was not feasible given the unexpected and rapid complete remission.

## Conclusion

We report a case of recurrent/metastatic PDAC in the abdominal wall with an exceptional treatment response on a novel combination immunochemotherapy including anti-LIF and anti-PD-L1 and standard-of-care chemotherapy. There was no evidence of a DNA repair deficiency in our patient’s PDAC, which could have accounted for the response to an immunotherapy-based protocol. Anti-LIF has been shown to reprogram immunosuppressive M2 TAMs into M1 antitumoral macrophages. The cancer tissue in our patient’s abdominal wall was highly enriched for TAMs that expressed CD163, a typical marker of immunosuppressive TAMs. We hypothesize that reprogramming those TAMs by anti-LIF into anti-tumoral M1 TAMs has contributed to the excellent and immediate treatment response. Despite the lack of an OS benefit of the combination of MSC-1 plus durvalumab and standard-of-care chemotherapy in this phase 2 first-line clinical trial compared with chemotherapy alone, we believe that this immunochemotherapy combination may have a role in certain PDAC patients, particularly those with cutaneous/subcutaneous metastatic disease.

## Data Availability

The original contributions presented in the study are included in the article/supplementary material. Further inquiries can be directed to the corresponding author.
